# Matrices

**Published:** 1885-02

**Authors:** 


### MATRICES.

For at least twelve years have we almost daily used the steel matrices alluded to in the report of the Northern Central Society of New Jersey, in filling approximal cavities between bicuspids and molars. It is no longer an experiment. After so long an experience with them one of two things is definitely established: either we are incompetent to properly judge of their merits, or to one whose methods of operating are anything like ours, they would prove almost indispensable when their action is fully comprehended. But they should not be used without thorough reflection as to their capabilities and disadvantages. In our early use of them it is probably the fact that teeth were sacrificed, because of inattention to certain rules that should be observed in their application.

Doubtless every operative dentist knows what they are, and their origin. The profession is indebted to Dr. Louis Jack, of Philadelphia, for the matrix, as it is for many other good things. Who first thrust a flat piece of steel between two teeth for the purpose of converting an approximal into a simple crown cavity, it would be hard to determine. Many dentists have used the safe side of a piece of separating file, but such a flat piece of steel will not answer the purpose of one of Jack's matrices, for the latter are carefully formed so as to restore the contour of a tooth instead of leaving a perfectly flat surface, and in a set of twelve may be found one that is adapted to any cavity on either side of the mouth. There are a number of grades of thickness, and one may obtain the "thin" "medium" or "thick." In most cases that require a matrix, we prefer the "thin." It is not within recollection that we have, in any case of ordinary decay of the proximal surfaces of bicuspids or molars, found it necessary to wedge the surfaces apart, other than what the matrix itself accomplishes, for the purpose of making space.

Every dentist knows that it is extremely difficult to insert fillings upon the posterior or distal surface of bicuspids and molars, especially if the cavities be near the cervical borders. When the teeth are crowded it is a painful and tedious process to gain the necessary space for filling in the usual way. The gold is not easily consolidated in such places either, and unless the most scrupulous care be exercised and the utmost skill be employed, there will be leaky places. But if the overlapping enamel be cut through, and the cavity made as large upon the grinding surface as anywhere in its extent, a number of points are gained.

First, every position of the decayed surface is brought into plain view, and it becomes possible to excavate perfectly ; secondly, the overlapping portion of coronal enamel being removed, there is less danger of the breaking down of the masticatory surface that proves fatal to many fillings ; and thirdly, it is possible to place the proper matrix between the two teeth and thus make a single cavity of the compound one, the operator being enabled not only to properly condense the gold upon the cervical wall and all through the cavity by a direct blow, but to perfectly restore the contour of the tooth in every point. There is another great advantage in this method of filling proximal cavities, and that is, no more gold than is necessary is introduced, and there is nothing to remove from between the teeth by files and burs. Emery paper and holly strips are quite sufficient for the perfect polishing of the gold.

Of course such an operation as this presupposes the existence of a tooth next the surface to be filled. But it is not essential that it should be very near ; if there be a moderate amount of space between them, two or more matrices may be inserted. If the space be considerable, a wedge of orange wood may be driven between two of the properly arranged matrices. In most cases it will be found necessary to drive a wedge against the matrix near the cervical border, to insure its close adaptation at that point. In filling a cavity so prepared, the utmost care should be observed in driving the gold toward the matrix and into the angle formed by the matrix and the edge of the tooth, at the margin of the cavity. This will be the point where most beginners in this method of filling will fail, and the fact cannot be too strongly impressed upon their minds. A little practice will make perfect, and those using this method will be delighted with the ease and perfection with which they can fill proximal cavities in the bicuspids and molars, *when all the margins are firm and strong*, and when the tooth is properly excavated. It must be remembered that there should be no undercuts to whose remotest point the plugger will not have direct access. The filling is to be held in place by making the cavity slightly retentive in shape upon the sides.

We said that this method of filling teeth would delight operators when the walls were perfect and strong. But if they be weak and friable the matrix is liable to prove a delusion and a snare. The operator finds it so easy to fill the ordinary proximal cavity when the tooth walls are firm, that he extends its use to weak, frail teeth, whose exposed edges need strengthening by judicious knuckling and protection. The gold in such cases should be carefully drawn over the exposed and fragile margins, and for this work the matrix is not adapted, except in a very small class of cases. The failures that have been encountered were in the injudicious use of this appliance where it was counter-indicated. A matrix was perhaps wedged up against infirm, frangible walls, and the gold then forcibly driven against them without being carried over sufficiently far to act as a protection. What wonder that these unprotected margins gave way, and the operation became a failure? The matrix is one of the most useful appliances that has been placed at the command of the dentist, but like the dental engine it should be used with judgment, else it may result in direct harm. In cases where some of the walls of an approximal cavity were gone, we have, however, frequently used a matrix ground down to one-half or less of its usual depth, and wedged the narrow blade against the cervical wall for the double purpose of holding the rubber down to place, and of supporting the foundation of the filling until past the point of principal danger. It also enables one to make sure of the adaptation of the gold at the most distant part of the filling.

In the insertion of plastic fillings in the distal surfaces of teeth, the matrix may be made to play a part even more important, if possible, than in the use of gold.

